# Immunothrombotic Dysregulation in Pediatric Patients Receiving Veno-arterial ECMO After Cardiac Surgery: Insights From Platelet Activation and T Cell Immune Profiling

**DOI:** 10.31083/RCM49903

**Published:** 2026-07-28

**Authors:** Junfeng Guo, Zhongtao Du, Bo Xu, Xiaofang Yang, Meng Xin, Xing Hao, Chenglong Li, Hong Wang, Xiaotong Hou

**Affiliations:** ^1^Department of Anesthesiology, Shengjing Hospital, China Medical University, 110801 Shenyang, Liaoning, China; ^2^Center for Cardiac Intensive Care, Beijing Anzhen Hospital, Capital Medical University, 100054 Beijing, China

**Keywords:** mechanical circulatory support, ECMO, platelets, thrombocytopenia, T lymphocytes

## Abstract

**Background:**

Numerous studies have demonstrated that both cardiopulmonary bypass (CPB) and extracorporeal membrane oxygenation (ECMO) are often associated with adverse outcomes or immune dysregulation when platelet counts decline during support. Moreover, the severity of thrombocytopenia generally correlates with a worse clinical prognosis. However, the underlying mechanisms remain incompletely understood. Previous research has attributed this phenomenon to hemodynamic alterations during CPB and ECMO, as well as anticoagulant-induced abnormal platelet activation. Based on our clinical observations, we question the completeness of this explanation and propose an alternative hypothesis: sustained platelet activation during ECMO serves as an initiating factor that contributes to both progressive thrombocytopenia and immune dysfunction throughout the support period.

**Methods:**

We investigated platelet activation status, degranulation capacity, and T cell subset dynamics in patients receiving ECMO support. Flow cytometry was used to assess platelet surface markers, reticulated platelet proportions, and T cell subset distributions.

**Results:**

We demonstrate that platelets exhibit excessive activation during ECMO, accompanied by a gradual decline in their degranulation capacity. Notably, even with an increased proportion of newly formed platelets, this functional impairment persists. Furthermore, we observed significant alterations in Helper T cell Type 1 (Th1) and CD8+ T cell populations during ECMO support.

**Conclusions:**

Our findings suggest that aberrantly activated platelets during ECMO amplify the specific recognition of platelet antigens by CD8+ T cells via modulating the differentiation bias of CD4+ T cells, particularly Th1 cells, ultimately leading to platelet depletion. These results suggest that early interventions targeting abnormal platelet activation may represent a potentially effective therapeutic strategy to mitigate ECMO-associated thrombocytopenia.

## 1. Introduction

Cardiopulmonary Bypass (CPB) and extracorporeal membrane oxygenation (ECMO) have become the most common basis for extracorporeal life support in clinical practice and are widely used in the clinical treatment of various types of critically ill patients. Since Gibbon’s [1] pioneering application of CPB in cardiac surgery during open-heart procedures in 1953, research efforts have increasingly focused on the miniaturization of extracorporeal systems and their adaptation for use beyond the operating room. This evolution facilitated the development of ECMO, which better meets these clinical demands and has been increasingly adopted in intensive care settings. Notably, veno-arterial ECMO (VA-ECMO) has emerged as a crucial therapeutic strategy for refractory cardiogenic shock and other acute cardiovascular conditions, providing life-saving support for patients with terminal cardiopulmonary failure or sudden hemo-dynamic instability [2,3]. Despite advancements in ECMO technology, thrombotic and hemorrhagic complications remain common and present significant clinical challenges [4,5].

Emerging evidence indicates that patients frequently develop thrombocytopenia, impaired platelet function, and immune dysregulation during ECMO support; however, the underlying mechanisms remain incompletely understood [6,7,8]. Our findings further demonstrate that patients experiencing a 50% or greater reduction in platelet count during ECMO therapy are at significantly increased risk of adverse clinical outcomes [9]. Additionally, our data reveal that platelet counts tend to stabilize at a reduced level between 3 to 5 days of ECMO support. Moreover, in clinical practice, we have observed that patients assisted by ECMO often experience immune dysfunction both during and after the ECMO support period.

Platelets are widely recognized as one of the key components involved in hemo-stasis and coagulation. However, recent studies have revealed that platelets also play a regulatory role in various immune responses [10,11,12]. Because of their anucleate structure, most of the genetic material is derived from megakaryocytes. As a result, an increasing number of researchers advocate for the integrated analysis and investigation of megakaryocytes and platelets. Moreover, accumulating evidence has demonstrated that platelets actively participate in modulating innate and adaptive immune responses across various pathological conditions through direct interactions with T cells.

To further elucidate the underlying etiology of thrombocytopenia in pediatric patients undergoing ECMO support and to investigate the potential pathophysiological mechanisms linking thrombocytopenia with immune dysregulation, we analyzed platelet phenotypes, markers of abnormal activation, and alterations in T-cell subsets at multiple time points during ECMO support. Our findings indicate that ECMO-associated thrombocytopenia results from the combined effects of aberrant platelet activation and dysregulated cellular immune responses.

## 2. Methods

### 2.1 Study Participants and Sample Preparation

Using a blood collection tube (with a blue cap) containing sodium citrate for anticoagulation, 2.7 mL of oxygenated peripheral blood was collected from the oxygenator of the ECMO circulation pipeline. The preparation of platelet-rich plasma (PRP) typically involves a two-step centrifugation protocol. In brief, citrate-anticoagulated whole blood was initially centrifuged at 200 g to separate the supernatant, which contains platelets and plasma. Subsequently, this supernatant was subjected to a second centrifugation at 2000 g to further remove residual plasma components. Finally, the resulting platelet pellet was resuspended in Hank’s Balanced Salt Solution (HBSS) buffer to obtain the PRP.

### 2.2 Flow Cytometry for Count of Platelets and the Content of Surface Glycoprotein Receptors (GP)

5 μL of citrate sodium anticoagulated whole blood sample was stained with pre-mixed antibody solution and incubated at room temperature in the dark for 15 minutes. All the antibodies required for this experiment are listed in **Supplementary Table 1**. After the incubation was completed, 500 μL of 1% polyformaldehyde solution was added to terminate the immune reaction. Then, the pre-diluted counting beads were added to the reaction system according to the experimental ratio and mixed well. Ths BD Canto Plus flow cytometer was then used for detection. Platelets were screened based on their forward scattering angle (FSC) and side scattering angle (SSC) characteristics, CD41+ and CD61+ were used for platelet labeling.

### 2.3 Platelet Activation Status Analysis

5 μL of whole blood was stimulated with and without adenosine diphosphate (ADP) (0.004 mmol/L) for 5 minutes in a dark environment and stained with anti-P-selectin-BV421 (BD Bioscience), anti-CD63 (Biolegend), anti-activated integrin GPIIb/IIIa (PAC-1, BD Bioscience) and anti-CD41-PE (Biolegend) for 15 min at room temperature (25 ± 5) °C. The reaction was terminated by adding an additional 500 μL of 1% paraformaldehyde solution, and data analysis was performed using the Canto plus flow cytometer within 6 hours. CD41+ was used for platelet labeling. All the antibodies required for this experiment are listed in **Supplementary Table 1**. For details on flow cytometry-related gating strategies refer to **Supplementary Material 2**.

### 2.4 Platelet RNA Analysis

The citrate sodium anticoagulated whole blood was incubated with anti-CD61-Alexa flour 647 and anti-HLA-A, B, C-BV510 in a dark environment for 15 minutes. Further 1% paraformaldehyde for fixation, and thiazole orange (TO) agent for staining were added for 5 minutes before loading onto the instrument and mixed thoroughly. The antibodies used are listed in **Supplementary Table 1**.

### 2.5 Apoptosis analysis

An apoptosis experiment was conducted using the Annexin V-FITC Apoptosis Detection Kit (Biolegend) and Fixable Viability Stain 660 (FVS660) Kit from BD Bioscience. According to the instructions, 200 μL of platelets were pre-separated and re-suspended in 1 × PBS. 1 μL of FVS660 staining solution was added and incubated at 4 °C in the dark for 5 minutes. This was washed twice with PBS containing 10% BSA, then Ca^2+^ and Mg^2+^-containing HBSS buffer was added, along with Annexin V and Alexa Fluor-labeled CD61 (AF-CD61) antibody. This was incubated in the dark for 15 minutes, washed twice with PBS, and then 500 μL of 1% paraformaldehyde was added to terminate the reaction. The reaction should be detected within 6 hours. The antibodies used are listed in **Supplementary Table 1**.

### 2.6 Statistics

Prism 9 (GraphPad Software Inc., San Diego, CA, USA) was employed for descriptive statistics, statistical analysis, and graphical representation. Due to the exploratory nature of this study and the limited sample size, data are presented as median with interquartile range (IQR). The Friedman test, a non-parametric alternative to repeated-measures analysis of variance (ANOVA), was employed to compare changes in platelet parameters and T-cell subsets across multiple time points (e.g., baseline, Day 1, Day 3, Day 5). Post-hoc pairwise comparisons between specific time points were conducted using the Wilcoxon signed-rank test. To control for the inflation of Type I error due to multiple comparisons, the Benjamini-Hochberg false discovery rate (FDR) correction was applied. A corrected *p*-value < 0.05 was considered statistically significant. It should be noted that, for clarity and visual readability, *p* values for nonsignificant results were omitted when they did not pertain to the experimental hypotheses. All analyses were considered exploratory and hypothesis-generating.

## 3. Results

### 3.1 ECMO Promotes Platelet Glycoprotein Defects and Abnormal Activation

**Fig. 1.**
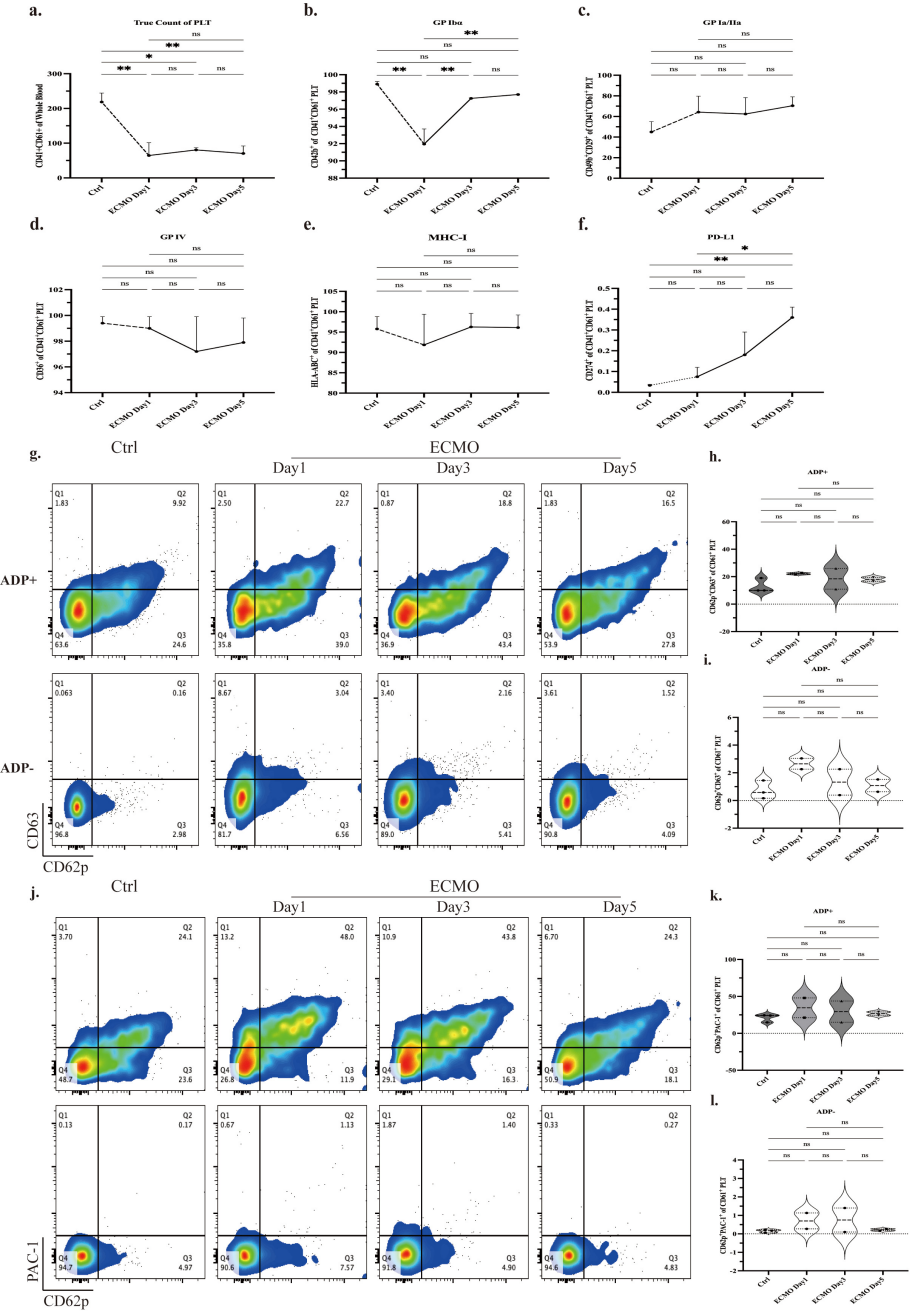
**Flow cytometry for the accurate counting of platelets**. (a). Dynamic changes on the surface of platelets of GP Iα (b). GP Ia/IIa (c). GP IV (d). HLA-A, B, C (e). and PD-L1 (f). The scatter plots and heat maps (g,j) show the monitoring of abnormal platelet activation (i,l) and ADP *in vitro* activation (h,k) during ECMO assistance at different times. (*p < 0.05; **p < 0.01; ***p < 0.001; ns, not significant.); Friedman’s test for comparison at respective time points. ANOVA, analysis of variance; ADP, adenosine diphosphate; ECMO, extracorporeal membrane oxygenation; GP, glycoprotein receptors.

**Fig. 2. Dynamic changes in reticulated platelet percentage and platelet apoptosis during ECMO support.**
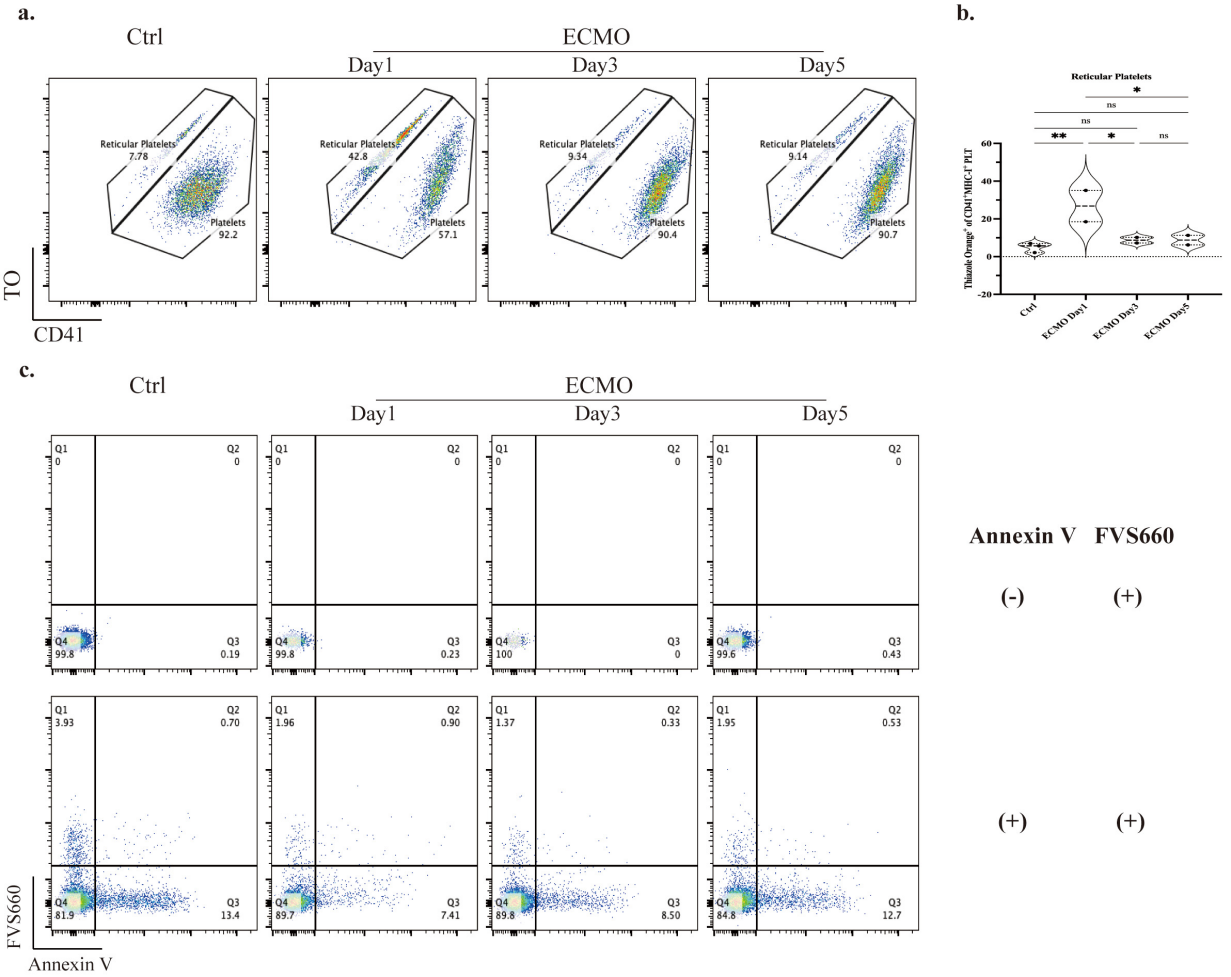
The scatter plot illustrates the dynamic changes in the proportion of reticulocytes in the bloodstream during various durations of ECMO support, as determined by flow cytometry (a,b), along with a schematic representation depicting the status of platelet apoptosis and structural damage (c). FVS660, Fixable Viability Stain 660; ECMO, extracorporeal membrane oxygenation. “*” represents *p* < 0.05, “**” represents *p* < 0.01, “ns” stands for “not significant”, i.e., *p* > 0.05.

This study enrolled two pediatric patients receiving VA-ECMO support following cardiac surgery (ECMO group), along with three matched control subjects selected based on age and surgical procedure (control group). Detailed information is provided in **Supplementary Material 1**. Based on previous research results, scholars generally believe that the occurrence of thrombocytopenia during ECMO assistance is closely related to abnormal platelet activation and/or mechanical injury. However, there is a lack of direct evidence. Initially, we applied flow cytometry technology to separately detect the platelet count and glycoprotein receptor content in the peripheral blood of ECMO-assisted children on the 1st, 3rd, and 5th days of assistance. We found that in pediatric patients, platelet count dropped sharply on the first day of ECMO assistance and remained at a low level throughout the 5-day period of assistance (Fig. 1a). Additionally, the platelet surface adhesion-related receptors GP Ibα and GP Ia/IIa complex, as well as GP IV (CD36) and immunoregulation-related receptors major histocompatibility complex class I (MHC-I, covering HLA-A, HLA-B and HLA-C) and programmed death-ligand 1 (PD-L1), all exhibit varying degrees of altered expression in the patient cohort (Fig. 1b–f), and significant reduction in GP Ibα was observed on the first day of assistance (Fig. 1b), which may be related to the strong platelet production capacity but poor membrane stability in children. Studies have shown that abnormal platelet glycoprotein receptors or abnormal activation can significantly enhance the clearance effect of the liver reticuloendothelial system, and this seems to be the most common cause of ECMO-related thrombo-cytopenia at present. To confirm this, we used TO to label and semi-quantify the RNA content of platelets. Platelets’ behavior, as cells without a nucleus, depends on the RNA inherited from megakaryocytes, and the content of RNA can also determine the lifespan of platelets [13,14]. We found that on the first day of ECMO assistance, the proportion of reticulocytes in the children’s circulation (with high RNA content) significantly increased and decreased to normal levels within 48 hours (Fig. 2a,b).

Next, we further assessed the activation status of platelets during ECMO support. By analyzing CD62p+CD63+ and CD62p+PAC-1+ platelet populations, we observed that the degree of circulating platelet activation was significantly elevated compared to the control group within the first five days of ECMO support (Fig. 1g,j). Although the activation levels showed a decreasing trend and gradually approached those of the control group starting from day 1, the differences remained statistically nonsignificant (Fig. 1i,l). Moreover, upon *in vitro* stimulation with ADP, the platelet activation response in the ECMO group exhibited a higher activation ratio than that in the control group; however, this difference did not reach statistical significance (Fig. 1h,k). The findings demonstrate that during ECMO support, platelets display functional receptor defects and abnormal activation patterns, which may play a role in the development of ECMO-associated thrombocytopenia. However, *in vitro* activation assays induced by ADP revealed a reduced activation reserve capacity in circulating platelets, a result that partially contradicts the proposed hypothesis.

### 3.2 ECMO Causes Mechanical Damage to Platelets

Furthermore, the hemorheological properties of blood flowing through the ECMO circuit, in combination with the mechanical shear force imposed by the vertical centrifugal pump, can directly impair platelet structural integrity. Due to their lack of a nucleus, platelets have limited capacity to respond to and repair mechanical damage. When the magnitude of injury exceeds their physiological tolerance, platelets become susceptible to apoptosis or necrosis. We found that regardless of whether ECMO assistance was performed or not and the duration of such assistance, cardiac surgery had a more significant impact on platelet damage (Fig. 2c).

### 3.3 Detection of T Cell Subsets and Activation Status

Platelets are the second most abundant cell type in human blood and exert various immune-regulatory functions under both physiological and pathological conditions. In fact, immune cell regulation via platelets has been demonstrated in several studies within the past decade [15]. Based on previous results, we observed that although platelet reduction, abnormal platelet activation, and glycoprotein deficiency occurred at the initiation of ECMO support in pediatric patients, these abnormalities were transient in nature. They were predominantly evident only on the first day of support and subsequently normalized to levels comparable to those of the control group. Nevertheless, platelet counts did not return to pre-support levels, indicating the presence of ongoing factors impairing platelet production. Integrating these observations with existing studies in adult populations [16], we hypothesize that this phenomenon may be associated with dysregulated cellular immunity.

**Fig. 3.**
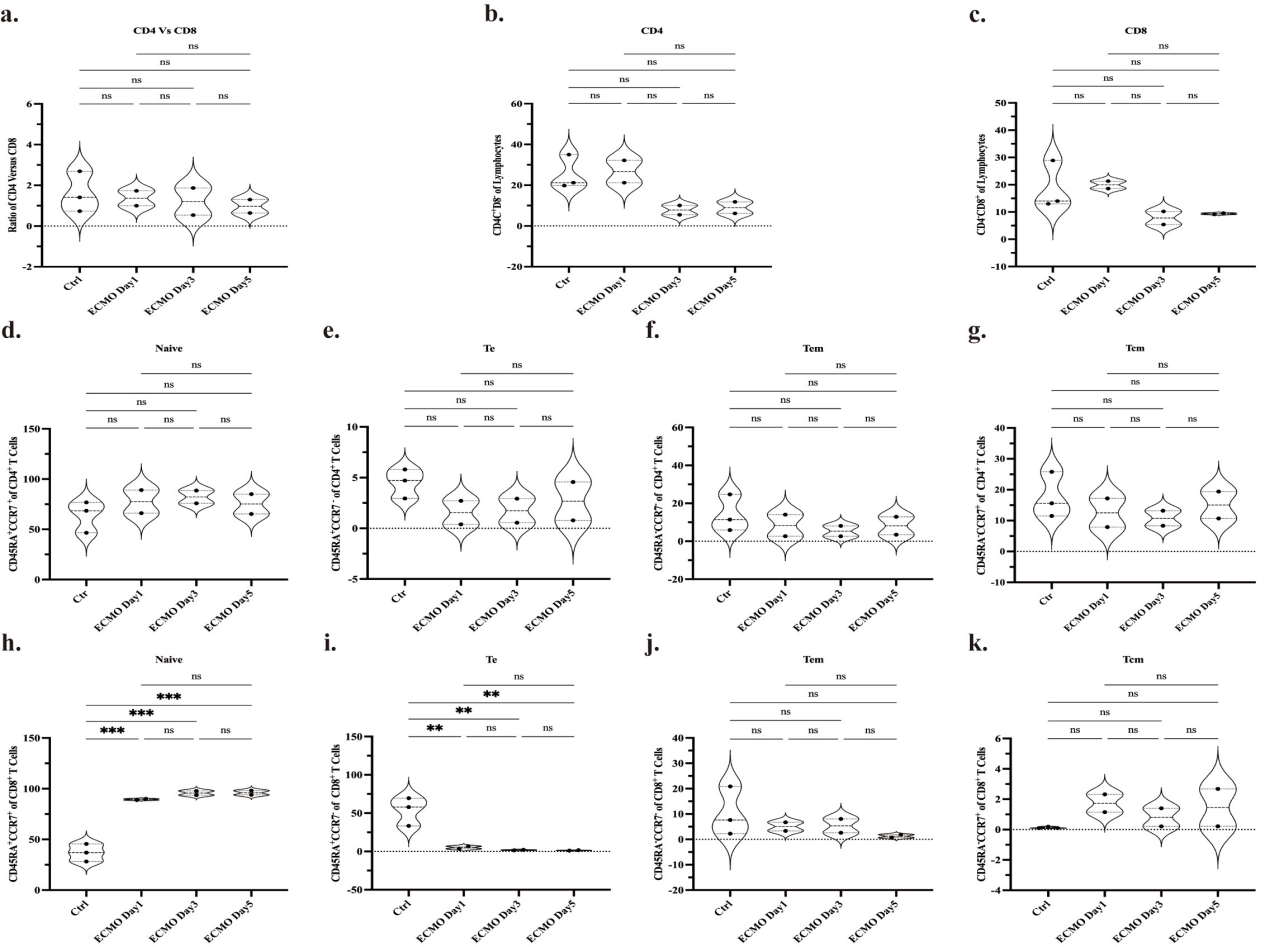
**Detection of T cell subset proportions and activation status**. (a) Illustrates the dynamic changes in the CD4/CD8 ratio at different time points during ECMO support; (b,c) depict the fluctuations in the proportions of CD4+ and CD8+ T cells, respectively; (d–g) demonstrate the variations in the proportions of naïve, effector, effector memory, and central memory CD4+ T cells. (h–k) illustrate the dynamic changes in the proportions of naïve, effector, effector memory, and central memory CD8+ T cells. ECMO, extracorporeal membrane oxygenation. “*” represents *p* < 0.05, “**” represents *p* < 0.01, “***” represents *p* < 0.001, “ns” stands for “not significant”, i.e., *p* > 0.05.

Therefore, we initially examined the proportions and activation status of peripheral blood T cell subsets at various time points during ECMO support. Our findings revealed a continuous decline in the CD4/CD8 ratio starting from the first day of support (Fig. 3a). Both CD4 and CD8 T cell proportions exhibited a transient increase on day 1, followed by a decrease to relatively low levels (Fig. 3b,c). Furthermore, analysis of T cell activation status demonstrated that, following the initiation of ECMO support, the proportion of naive cells (CD45RA+CCR7+) within both CD4 and CD8 T cell populations was markedly higher compared to the control group (Fig. 3d,h). Conversely, the proportions of effector cells (CD45RA+CCR7-), effector memory cells (CD45RA-CCR7-), and central memory cells (CD45RA-CCR7+) were all reduced to varying extents relative to the control group (Fig. 3e–g,i–k). Notably, the proportion of effector CD8 T cells were significantly decreased (Fig. 3i), showing statistically significant differences (*p* < 0.05). These findings indicate that specific factors related to ECMO support may induce T cell immaturity and suppress their activation and proliferation, thereby contributing to the disruption of immune homeostasis.

### 3.4 Detection of Helper T Cell Subsets (Th)

Research demonstrates that CD63 maintains the quiescent state of hematopoietic stem cells (HSC) and suppresses T cell proliferation and activation via the TGF-beta signaling pathway [15,17]. Our research findings further demonstrate that the expression level of PD-L1 on the surface of circulating platelets in patients, progressively increases during ECMO support (Fig. 1f). Subsequently, we hypothesize that platelets over-activated during ECMO support may contribute to the disruption of T cell homeostasis. For this purpose, we also analyzed the subgroups of helper T cells, which are the most important components of the cellular immune process.

**Fig. 4.**
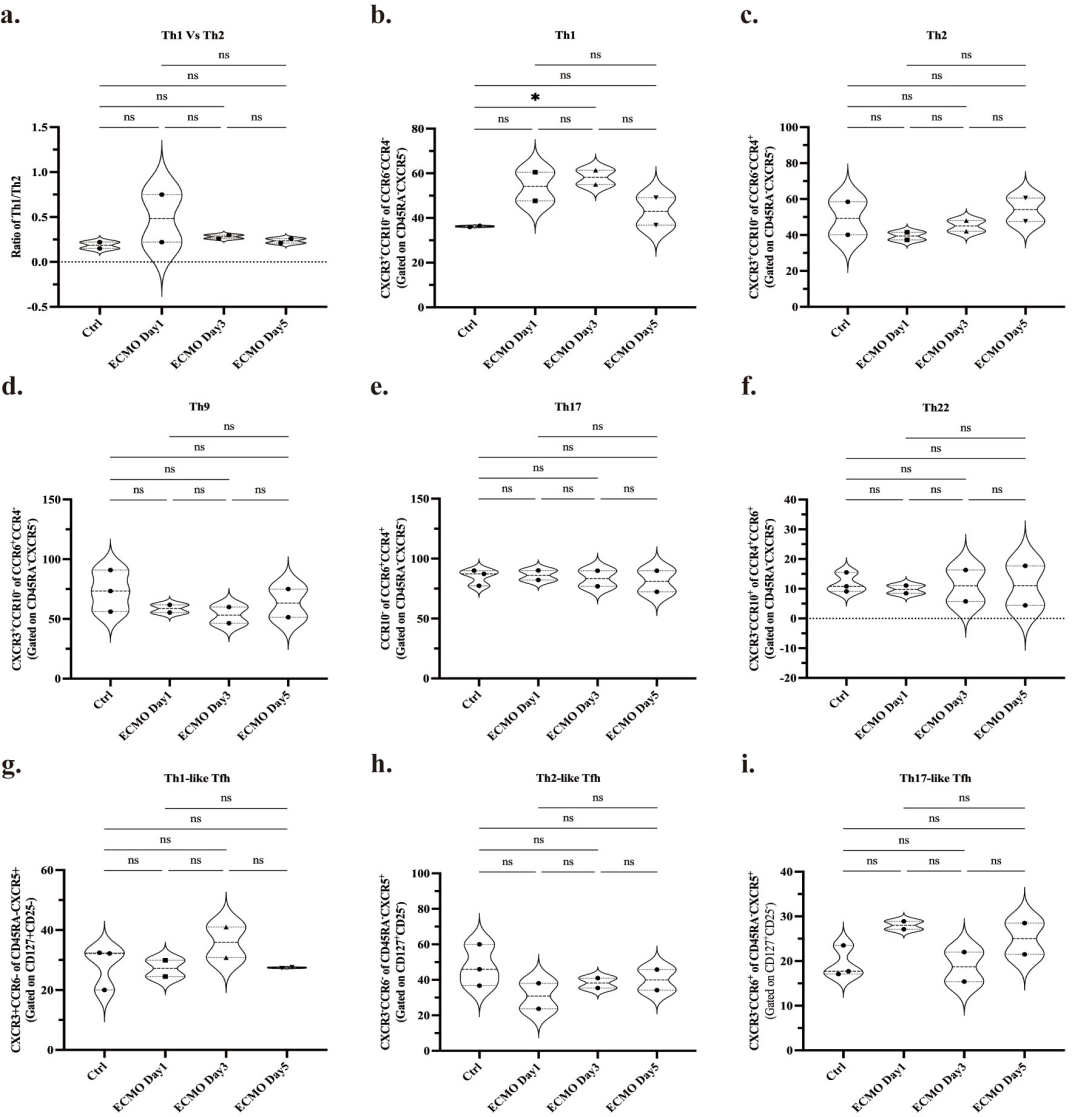
**Changes in the proportions of T cell subsets detected by flow cytometry**. Flow cytometry for the Th1/Th2 ratio (a) at various time points during ECMO support, along with the proportions of Th1 (b), Th2 (c), Th9 (d), Th17 (e), and Th22 (f) helper T cell subsets, as well as the frequencies of Th1-like (g), Th2-like (h), and Th17-like (i) follicular helper T cells. ECMO, extracorporeal membrane oxygenation. “*” represents *p* < 0.05, “**” represents *p* < 0.01, “***” represents *p* < 0.001, “ns” stands for “not significant”, i.e., *p* > 0.05.

Using flow cytometry, we observed distinct dynamic changes among Th cell subpopulations at different time points during ECMO support. The Th1/Th2 ratio showed a significant increase only on the first day of support and returned to levels comparable to those of the control group by the third day (Fig. 4a). In contrast to the Th1/Th2 ratio, the proportion of Th1 cells in peripheral blood was markedly elevated at the initiation of ECMO support and further increased by day three (Fig. 4b, *p* < 0.05). Th1 cells play a pivotal role in cellular immunity, particularly in promoting the proliferation and maturation of CD8+ T cells. An elevated proportion of Th1 cells has also been associated with enhanced induction of delayed-type hypersensitivity reactions (DTH). Meanwhile, the proportions of Th2 and Th9 cells gradually declined as support duration extended (Fig. 4c,d). No notable changes were observed in the frequencies of Th17 and Th22 cells throughout the observation period (Fig. 4e,f). Compared with conventional helper T cells, the dynamic patterns of follicular helper T cells (Tfh) were largely consistent with previously reported adult profiles (unpublished data from our center), apart from a pronounced increase in Th17-like Tfh cells (Fig. 4g–i). Collectively, these findings indicate a strong association between thrombocytopenia during ECMO support and both abnormal platelet activation and T-cell dysregulation.

## 4. Discussion

Platelets are generated by megakaryocytes (MKs) through budding or cytoplasmic fragmentation. Under physiological conditions, their production and clearance are tightly regulated by immune mechanisms and the hepatic reticuloendothelial system. Given their unique anuclear structure, platelets inherit nearly all their functional properties from MKs [18,19]. However, emerging evidence suggests that the distinct polyploid nuclear architecture of megakaryocytes contributes to substantial functional heterogeneity among the platelets they produce. Clinical observations reveal a temporal correlation between platelet count reduction and immune dysfunction during ECMO support, although the underlying mechanisms remain incompletely understood. Previous studies have primarily attributed ECMO-associated thrombocytopenia to anticoagulant use and abnormal platelet activation caused by exposure to non-biological surfaces [16,20,21,22], yet immune dysregulation has not been systematically investigated. To our knowledge, this study is the first to explore the potential link between ECMO-related thrombocytopenia and immune dysfunction, offering insights into why thrombocytopenia predominantly occurs during ECMO support.

Under physiological conditions, P-selectin (CD62p) is predominantly localized in platelet α-granules, whereas CD63 resides on the lysosomal/dense granule membrane and serves as both a key marker of late platelet activation and a mediator of immune regulation [10,17,23]. Our experimental data from platelet activation and *in vitro* stimulation assays demonstrate that during ECMO support, platelets exhibit varying degrees of abnormal activation and display heterogeneous responses to ADP stimulation. Notably, the proportion of CD62p+CD63+ platelets was significantly elevated compared to the control group, while the increase in CD62p+PAC-1+ platelets remained minimal. Platelets originate from megakaryocytes with diverse ploidy levels, resulting in functional heterogeneity among them. Specifically, platelets derived from low-ploidy megakaryocytes (<8N) not only participate in coagulation and also contribute to the modulation of various immune responses. Intriguingly, upon *in vitro* ADP stimulation, platelets from ECMO-supported patients failed to show the expected significant increase in activation levels, suggesting the presence of platelet aging, which may be associated with excessive degranulation induced by hemodynamic alterations during extracorporeal circulation.

Increasing evidence demonstrates that platelets not only serve as critical regulators and mediators in the coagulation process, they also actively participate in various immune and inflammatory responses, including tumor immune escape [24], the pathogenesis of autoimmune diseases [25], and the development of systemic inflammatory response syndrome [26], via signaling pathways including PD-L1/PD-1 and TGF-β.

Regarding cellular immune responses, previous clinical observations have indicated that adult patients receiving ECMO support typically exhibit a decline in platelet counts within 3–5 days of support initiation, a finding consistent with our data. This phenomenon occurs exclusively during ECMO support. Following ECMO discontinuation, platelet counts generally return to near-baseline levels within 72 hours without pharmacological intervention. Notably, the initiation of classical delayed-type hypersensitivity also occurs approximately 72 hours after antigen exposure and depends on persistent antigen stimulation. Based on this temporal correlation, we hypothesize a potential link between ECMO-associated thrombocytopenia and the kinetics of delayed-type hypersensitivity. To investigate, we monitored CD8+ T cells-the key effector cells in delayed-type hypersensitivity-and observed a progressive decrease in the proportion of effector CD8+ T cells and central memory CD8-positive T cells, alongside a gradual increase in effector memory CD8-positive T cells. Concurrently, we noted a significant rise in the proportion of Th1 cells, which assist in CD8+ T cell proliferation and activation, during ECMO support. This finding may be associated with ECMO-induced platelet activation interfering with regulatory T cell (Treg) function [20]. The observed trend aligns with our hypothesis and further suggests that aberrant platelet activation during ECMO may suppress CD4+ T cell function, thereby enhancing CD8+ T cell activity within a certain range and affecting polyploid megakaryocytes. This mechanistic hypothesis is additionally supported by experimental observations of upregulated PD-L1 expression on platelet surfaces during ECMO support.

In conclusion, the data from this study indicate that abnormally activated platelets during ECMO support may serve as an initiating factor, playing a critical role in promoting the occurrence and progression of thrombocytopenia and immune dysregulation. Therefore, whether interference with or suppression of abnormal platelet activation can prevent or correct immune dysregulation—thereby aiding in the prevention and treatment of ECMO-associated thrombocytopenia—represents a pivotal issue that warrants further in-depth investigation.

## 5. Limitations

While we have drawn our research conclusions, it is important to acknowledge that our study still has several limitations. First, due to the small sample size (n = 5), the results may be subject to bias. Expanding the cohort in future studies will be essential to enhance the reliability and generalizability of the findings. Furthermore, this study did not evaluate the differential effects of various types and doses of anticoagulants on pediatric patients receiving ECMO support. A well-designed randomized controlled trial (RCT) is urgently warranted to address this gap.

## 6. Conclusions

In conclusion, this study demonstrates that sustained platelet hyperactivation during ECMO support triggers a cascade of functional and immunological alterations. We observed that excessive platelet activation is accompanied by progressive degranulation impairment, which persists even with increased production of newly formed platelets. Importantly, our findings reveal a novel mechanistic link between aberrant platelet activation and T-cell-mediated immune responses: platelet hyperactivation promotes Th1 cell differentiation bias, which in turn enhances CD8+ T-cell recognition of platelet antigens, ultimately contributing to platelet depletion. These results challenge the traditional view that merely attributes ECMO-associated thrombocytopenia to hemodynamic changes or anticoagulant effects. Instead, we propose that platelet activation serves as an initiating and amplifying factor in both thrombocytopenia and immune dysregulation. Early therapeutic interventions specifically targeting abnormal platelet activation may therefore represent a promising strategy to mitigate ECMO-related complications and improve patient outcomes.

## Data Availability

Regarding the availability of data and materials, there are currently no additional supporting files, and interested readers may contact the corresponding author for relevant requests.

## Abbreviations

VA-ECMO, Veno-arterial extracorporeal membrane oxygenation; CPB, cardiopulmonary bypass.

## References

[1] Gibbon JH (1954). Application of a mechanical heart and lung apparatus to cardiac surgery. Minnesota Medicine.

[2] Vyas A, Bishop MA (2022). Extracorporeal Membrane Oxygenation in Adults.

[3] Friedrichson B, Mutlak H, Zacharowski K, Piekarski F (2021). Insight into ECMO, mortality and ARDS: a nationwide analysis of 45,647 ECMO runs. Critical Care (London, England).

[4] Aubron C, DePuydt J, Belon F, Bailey M, Schmidt M, Sheldrake J (2016). Predictive factors of bleeding events in adults undergoing extracorporeal membrane oxygenation. Annals of Intensive Care.

[5] Doyle AJ, Hunt BJ, Sanderson B, Zhang J, Mak SM, Benedetti G (2021). A Comparison of Thrombosis and Hemorrhage Rates in Patients With Severe Respiratory Failure Due to Coronavirus Disease 2019 and Influenza Requiring Extracorporeal Membrane Oxygenation. Critical Care Medicine.

[6] Tanabe M, Hosokawa K, Nguyen MAT, Nakagawa N, Maruyama K, Tsuji N (2022). The GPI-anchored protein CD109 protects hematopoietic progenitor cells from undergoing erythroid differentiation induced by TGF-β. Leukemia.

[7] Goudswaard LJ, Williams CM, Khalil J, Burley KL, Hamilton F, Arnold D (2023). Alterations in platelet proteome signature and impaired platelet integrin α_IIb_β_3_ activation in patients with COVID-19. Journal of Thrombosis and Haemostasis : JTH.

[8] Mansour A, Roussel M, Gaussem P, Nédelec-Gac F, Pontis A, Flécher E (2020). Platelet Functions During Extracorporeal Membrane Oxygenation. Platelet-Leukocyte Aggregates Analyzed by Flow Cytometry as a Promising Tool to Monitor Platelet Activation. Journal of Clinical Medicine.

[9] Wang L, Shao J, Shao C, Wang H, Jia M, Hou X (2021). The Relative Early Decrease in Platelet Count Is Associated With Mortality in Post-cardiotomy Patients Undergoing Venoarterial Extracorporeal Membrane Oxygenation. Frontiers in Medicine.

[10] Vrbensky JR, Nazy I, Clare R, Larché M, Arnold DM (2022). T cell-mediated autoimmunity in immune thrombocytopenia. European Journal of Haematology.

[11] Guo L, Shen S, Rowley JW, Tolley ND, Jia W, Manne BK (2021). Platelet MHC class I mediates CD8+ T-cell suppression during sepsis. Blood.

[12] Rawish E, Nording H, Münte T, Langer HF (2020). Platelets as Mediators of Neuroinflammation and Thrombosis. Frontiers in Immunology.

[13] Noh JY (2021). Megakaryopoiesis and Platelet Biology: Roles of Transcription Factors and Emerging Clinical Implications. International Journal of Molecular Sciences.

[14] Gieger C, Radhakrishnan A, Cvejic A, Tang W, Porcu E, Pistis G (2011). New gene functions in megakaryopoiesis and platelet formation. Nature.

[15] Polasky C, Wendt F, Pries R, Wollenberg B (2020). Platelet Induced Functional Alteration of CD4^+^ and CD8^+^ T Cells in HNSCC. International Journal of Molecular Sciences.

[16] Rauch A, Dupont A, Rosa M, Desvages M, Le Tanno C, Abdoul J (2023). Shear Forces Induced Platelet Clearance Is a New Mechanism of Thrombocytopenia. Circulation Research.

[17] Hu M, Lu Y, Wang S, Zhang Z, Qi Y, Chen N (2022). CD63 acts as a functional marker in maintaining hematopoietic stem cell quiescence through supporting TGFβ signaling in mice. Cell Death and Differentiation.

[18] Roeser A, Moulis G, Ebbo M, Terriou L, Poullot E, Lioger B (2022). Characteristics, management and outcome of acquired amegakaryocytic thrombocytopenia. British Journal of Haematology.

[19] Liu C, Wu D, Xia M, Li M, Sun Z, Shen B (2021). Characterization of Cellular Heterogeneity and an Immune Subpopulation of Human Megakaryocytes. Advanced Science (Weinheim, Baden-Wurttemberg, Germany).

[20] Revel-Vilk S, Naamad M, Frydman D, Freund MR, Dinur T, Istaiti M (2022). Platelet Activation and Reactivity in a Large Cohort of Patients with Gaucher Disease. Thrombosis and Haemostasis.

[21] Klaeske K, Dieterlen MT, Eifert S, Scholz U, Garbade J, Jawad K (2021). Device-induced platelet dysfunction in patients after left ventricular assist device implantation. Journal of Thrombosis and Haemostasis : JTH.

[22] Genty T, Burguburu S, Imbert A, Roman C, Camille W, Thès J (2023). Circuit change during extracorporeal membrane oxygenation: single-center retrospective study of 48 changes. Critical Care (London, England).

[23] Li WJ, Peng YX, Zhao LQ, Wang HY, Liu W, Bai K (2024). T-cell lymphopenia is associated with an increased infecting risk in children after cardiopulmonary bypass. Pediatric Research.

[24] Paletta A, Di Diego García F, Varese A, Erra Diaz F, García J, Cisneros JC (2022). Platelets modulate CD4^+^ T-cell function in COVID-19 through a PD-L1 dependent mechanism. British Journal of Haematology.

[25] Manfredi AA, Ramirez GA, Godino C, Capobianco A, Monno A, Franchini S (2022). Platelet Phagocytosis via P-selectin Glycoprotein Ligand 1 and Accumulation of Microparticles in Systemic Sclerosis. Arthritis & Rheumatology (Hoboken, N.J.).

[26] Johnson JE, McGuone D, Xu ML, Jane-Wit D, Mitchell RN, Libby P (2022). Coronavirus Disease 2019 (COVID-19) Coronary Vascular Thrombosis: Correlation with Neutrophil but Not Endothelial Activation. The American Journal of Pathology.

