# Supplementary material for: Immunothrombotic Dysregulation in Pediatric Patients Receiving Veno-arterial ECMO After Cardiac Surgery: Insights From Platelet Activation and T Cell Immune Profiling
Source: Rev Cardiovasc Med. 2026 Jul 28;27(7):49903. doi: 10.31083/RCM49903 (PMC13419992; doi:10.31083/RCM49903)
Supplement: Supplementary file 1 [file 2153-8174-27-7-49903-s1.zip › Supplementary Material_Table_1 .docx]

**Flow cytometry**

Whole blood cells were stained with conjugated fluorescein antibody for 15min, and samples were fixed with 1% paraformaldehyde after lysis of erythrocytes. Flow cytometric analysis were conducted on a FACS Canto plus flow cytometer (BD Biosciences). Data were analyzed with FlowJo (version 10.8.1) software.

| Table S1. Antibody information for the flow cytometry. | | | | |
| --- | --- | --- | --- | --- |
| Markers |  | Clone | Cat # | Source |
| CD3 | PE | UCHT1 | 300408 | Biolegend |
| CD4 | APC-Fire750 | SK3 | 344638 | Biolegend |
| CD8 | PerCP-Cy5.5 | SK1 | 565310 | BD Bioscience |
| CD127 | PE-Cy7 | A019D5 | 351320 | Biolegend |
| CD25 | Alexa Fluor® 700 | M-A251 | 356118 | Biolegend |
| CD45RA | Brilliant Violet 510 | HI100 | 563031 | BD Bioscience |
| CD185 | Brilliant Violet 605 | J252D4 | 356930 | Biolegend |
| CD183 | FITC | G025H7 | 353704 | Biolegend |
| CD196 | PerCP-Cy5.5 | G034E3 | 353406 | Biolegend |
| CD194 | Brilliant Violet 421 | L291H4 | 359414 | Biolegend |
| CCR10 | APC | 6588-5 | 341506 | Biolegend |
| CD197 | Brilliant Violet 421 | G043H7 | 353208 | Biolegend |
| CD41 | PE | HIP8 | 303706 | Biolegend |
| CD61 | Alexa Fluor® 647 | VI-PL2 | 336408 | Biolegend |
| CD49b | PE-Cy7 | [PIE6-C5](https://www.biolegend.com/en-us/search-results?Clone=P1E6-C5) | 359314 | Biolegend |
| CD29 | APC-Cy7 | TS2/16 | 303014 | Biolegend |
| CD36 | APC | 5-271 | 336208 | Biolegend |
| HLA-ABC | Brilliant Violet 510 | W6/32 | 311436 | Biolegend |
| PD-L1 | FITC | MIH2 | 374510 | Biolegend |
| CD62P | Brilliant Violet 421 | AK4 | 304926 | Biolegend |
| CD63 | PE-Cy7 | H5C6 | 353010 | Biolegend |
| PAC-1 | FITC | PAC-1 | 340507 | BD Bioscience |
| CD42b | Brilliant Violet 421 | HIP1 | 303930 | Biolegend |
