# Supplementary figures and images for: Immunothrombotic Dysregulation in Pediatric Patients Receiving Veno-arterial ECMO After Cardiac Surgery: Insights From Platelet Activation and T Cell Immune Profiling
Source: Rev Cardiovasc Med. 2026 Jul 28;27(7):49903. doi: 10.31083/RCM49903 (PMC13419992; doi:10.31083/RCM49903)

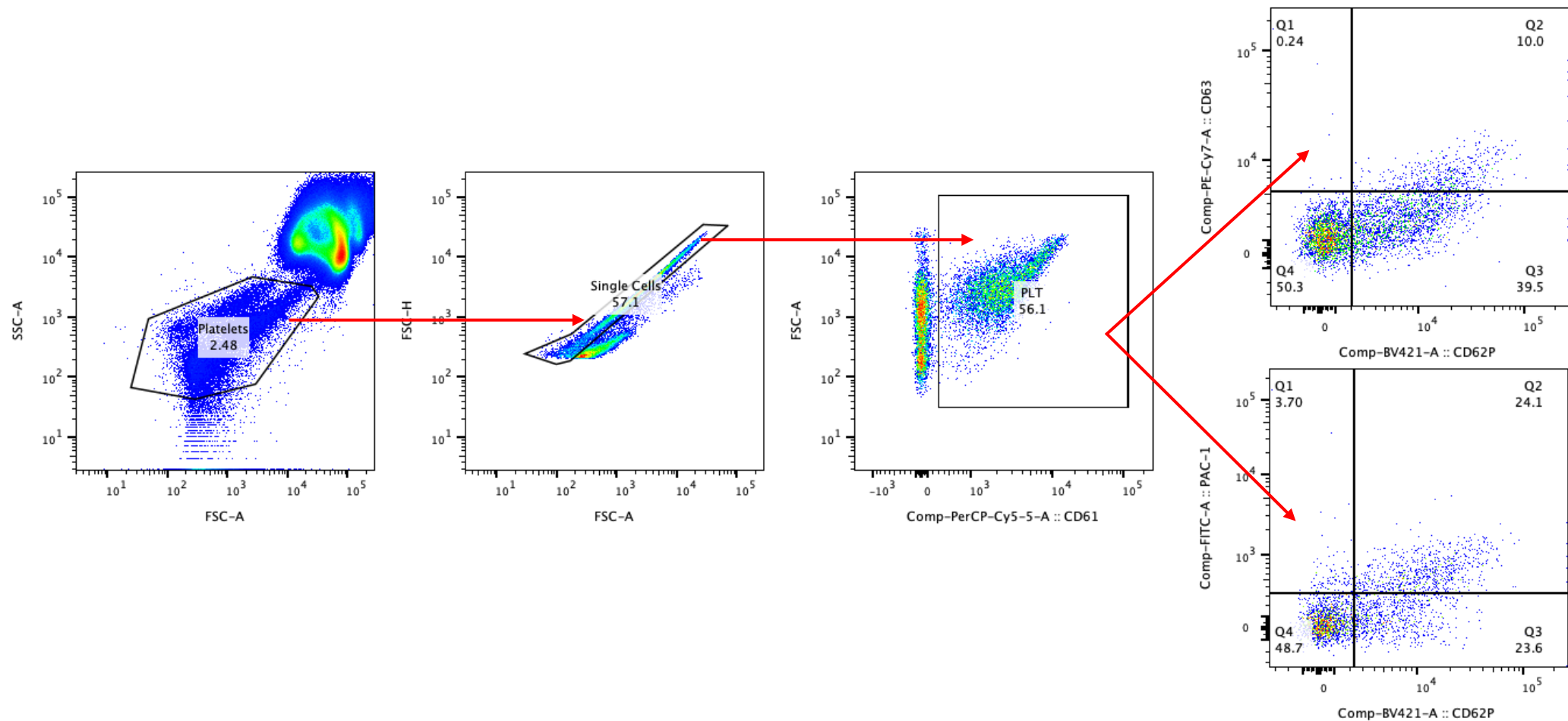

The relevant gating strategies for flow cytometry.

Supplement: Supplementary file 1 [file 2153-8174-27-7-49903-s1.zip › Supplementary Material 2.pdf]
